# A Reevaluation of the Factor Structure, Reliability, and Validity of the Spiritual Well-Being Questionnaire (SWBQ)

**DOI:** 10.1007/s10943-022-01619-0

**Published:** 2022-08-05

**Authors:** Rapson Gomez, Shaun Watson

**Affiliations:** Institute of Health and Wellbeing, Federation University Australia, University Drive, Mt Helen, PO Box 663, Ballarat, VIC 3353 Australia

**Keywords:** Spiritual Well-Being Questionnaire, SWBQ, Factor structure, Adults, Confirmatory factor analysis, Exploratory structural equation modeling

## Abstract

The 20-item Gomez and Fisher (Personal Individ Differ 35:1975–1991, 2003) Spiritual Well-Being Questionnaire (SWBQ) is a widely used measure of spiritual well-being. Its theoretical model is a higher-order model with primary factors for personal, communal, environmental, and transcendental well-being, and a secondary global spiritual well-being factor. The current study, conducted in Australia, reevaluated the factor structure of the SWBQ. Unlike previous studies, the current study also used exploratory structural equation modeling (ESEM) to examine the factor structure of the SWBQ and selected the preferred model using not only global model fit values, but also the clarity, reliabilities, and validities of the factors in the models. A total of 227 adults (males = 63; females = 164; *M* age = 26.1 years; SD = 5.2 years) completed the SWBQ. Based on the model selection criteria applied in the study, the ESEM model with four group factors was selected as the preferred model. However, there was also adequate support for the proposed theoretical higher-order model and the first-order oblique model with the four well-being factors. Concerning our preferred model, its factors showed reasonable clarity for factor loadings and (omega) reliabilities. However, only the communal domain scale was supported empirically for external validity. The implications of the findings for the theoretical model, the use of the SWBQ, and future studies are discussed. In this respect, there are three potential models (theorized higher-order model, 4-factor first-order oblique model, and the ESEM model proposed in this study) that warrant further detailed investigation with a larger, more representative population and additional validation measures.

## Introduction

According to the National Interfaith Coalition on Aging (1975), spiritual well-being is the affirmation of a life relationship with oneself (personal; or beliefs and perceptions about one’s own existence), others (communal; or relatedness with other people and community), nature (environment; or a relationship with environment and nature), and God (beliefs and deep relations with a greater and higher power, or transcendental other). Based on this, Fisher (1998) proposed a hierarchical multidimensional model of spiritual well-being, comprising four obliquely related primary factors (personal, communal, environmental, and transcendental) that cohere to form a higher-order global spiritual well-being factor. To measure these factors, Gomez and Fisher (2003) published the Spiritual Well-Being Questionnaire (SWBQ). With 20 items, it has scales for personal (items relating to intra-relations with oneself concerning meaning, purpose, and values in life), communal (items relating to the quality and depth of inter-personal relationships, between self and others, and includes love, justice, hope, and faith in humanity), environment (items relating to the individual’s relationship with the environment and nature, including a sense of awe, wonder, and unity with the environment), and transcendental other (items relating to the individual’s beliefs and deep relations with a greater and higher power and some-thing or some-one beyond the human level, such as God, and involves faith toward, adoration, and worship of the source of mystery of the universe). Corresponding to Fisher’s hierarchical spiritual well-being model, the theoretical model proposed for the SWBQ is a higher-order factor model, with factors for these four domains, and an overall global (general) spiritual well-being factor (total score for all items).

The SWBQ has become a popular measure for research on spiritual well-being. By 2016, it was translated into 27 languages and reported to have been used in over 500 studies internationally (Abhari et al., 2018). The SWBQ has been at times referred to as the lived experience component of a measure called the Spiritual Health and Life-Orientation Measure (SHALOM; Fisher, 2010). The SHALOM has two sets of 20 items identical to the SWBQ items. For the first item set, respondents are asked to indicate what they think are the ideal levels for the descriptors in them (the “ideal” component), and for the second item set, they are asked to indicate how the descriptors in the items describe their own experience (the “lived experience” component) over the last 6 months. Consequently, the psychometric properties of the lived experience component in the SHALOM are also applicable to the SWBQ. Given the focus of the current study, we will cover the psychometric literature relevant to the original SWBQ, and the lived experience component of SHALOM. For convenience, we refer to both sets as SWBQ.

A comprehensive review by de Jager Meezenbroek et al. (2012) that evaluated the psychometric qualities (psychometric properties, item formulation, and confusion with well-being and distress) of ten commonly used spirituality questionnaires (including the SWBQ) concluded that the SWBQ was the most promising measure. Indeed, they concluded that the SWBQ was the only measure with proven construct validity. The review also noted that for only one (“developing joy in life”) of its 20 items was there confusion with (health) well-being. Consequently, scores for the spirituality constructs in this measure and other health well-being measures will not be inflated due to tautology (in this case, the inclusion of health well-being items in the SWBQ and the other questionnaires measuring well-being). Overall, therefore, the SWBQ is well suited for research studies on spiritual well-being, including its relations with health, psychological, emotional, and general well-being. Indeed, a recent comprehensive review of the validation and utilization of the SHALOM concluded that it is a substantial measure of spiritual well-being (Fisher, 2021).

However, despite such positive qualities, as will be covered in detail, an examination of past confirmatory factor analysis (CFA) studies that have examined the factor structure of the SWBQ in terms of currently accepted standards for evaluating global model fit (Hu & Bentler, 1999) does not appear to concur with this conclusion. Consequently, the major aim of the current study was to reexamine the factor structure of the SWBQ. Unlike previous studies that used CFA, the current study examined the factor structure using the recently developed and advanced exploratory structural equation modeling approach (ESEM; Asparouhov & Muthén, 2009).

### Initial Development of the SWBQ

In the initial scale development study, Gomez and Fisher (2003) reported four empirical studies. In Study 1 (adolescent sample), they used principal component analysis to examine the factor structure of a preliminary version of the SWBQ (PSWBQ) that had 48 items (12 items in each domain). The findings supported the four-factor oblique structure, from which they developed the final version of the SWBQ by selecting the five highest loading items for each domain. In Study 2 (adolescent sample), EFA not only confirmed the four-factor oblique structure but also supported a second-order factor model, with one second-order general spiritual well-being factor and the theorized four primary factors. This model corresponds to Fisher’s (1998) hierarchical multidimensional model of spiritual well-being.

### Confirmatory Factor Analysis Studies of the Factor Structure of the SWBQ

**Fig. 1:**
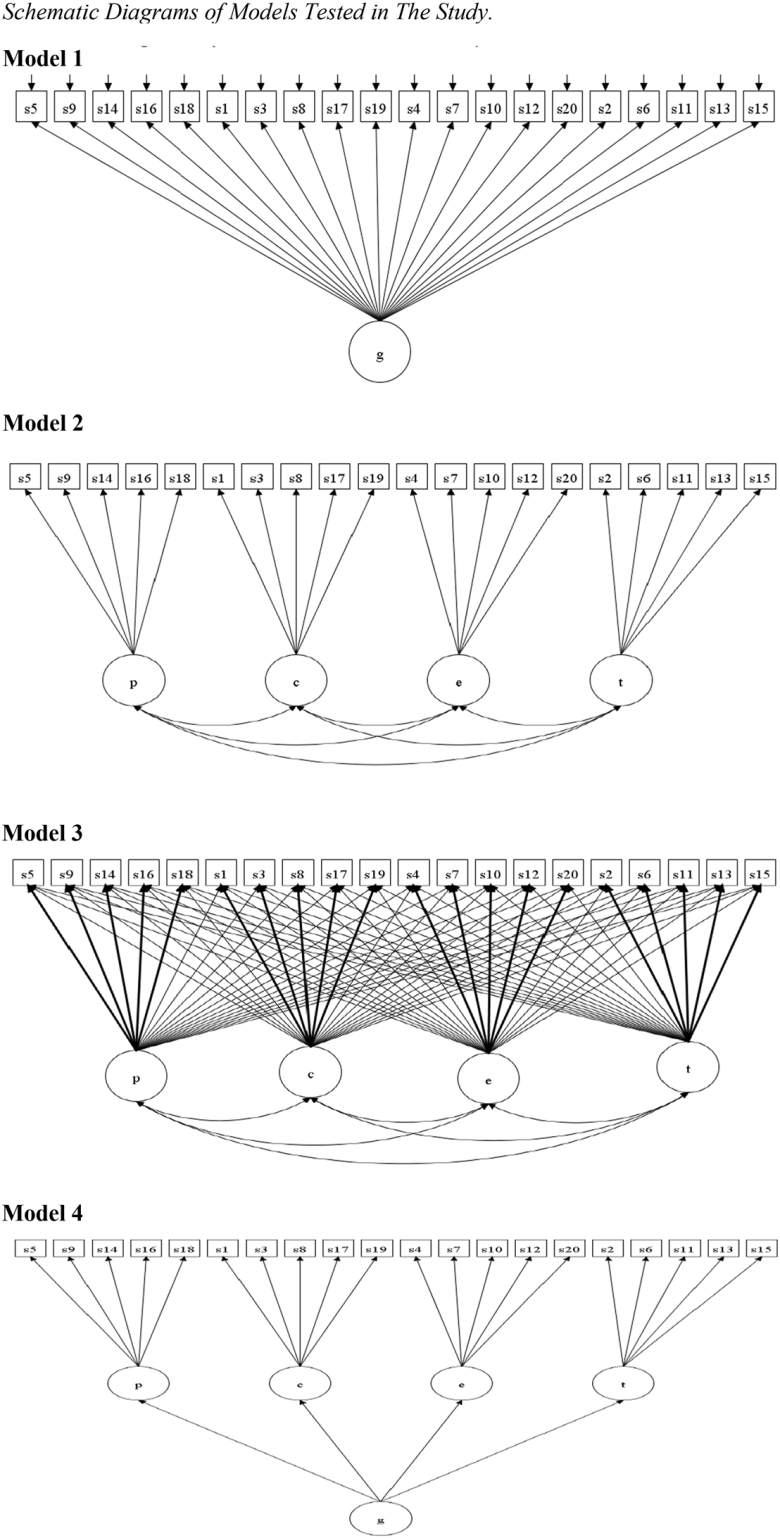
Schematic Diagrams of Models Tested in The Study. Model 1: One-factor model. Model 2: CFA four-factor oblique model. Model 3: ESEM with four oblique factors. Model 4: Higher-order factor model. For Model 3, bold arrows indicated designated items

To date, apart from the initial scale development study of the SWBQ (Gomez & Fisher, 2003), there have been other studies that have examined the four-factor structure of the SWBQ (Abhari et al., 2018; Elhai et al., 2018; Gomez & Fisher, 2005; Gouveia et al., 2012; Nunes et al., 2018; Pong et al., 2020; Rowold, 2011). Many of these studies have also examined a one-factor model (all 20 items loading on a single factor), a CFA four-factor orthogonal model (four uncorrelated factors), and a higher-order factor model (the four primary factors loading on a single higher-order factor). These models are depicted in Fig. 1.

With CFA, at the statistical level, global model fit is evaluated in terms of chi-square, with a nonsignificant value interpreted as indicative of a good fit. However, as chi-square values are inflated when sample sizes are large (as is often the case when studies conduct CFA), researchers generally use other approximate fit indices to evaluate global model fit. The commonly used indices in studies involving the SWBQ have been the root mean square error of approximation (RMSEA), the standardized root mean square residual/root mean square residual (SRMR/RMR), the comparative fit index (CFI), and the goodness of fit index (GFI). According to the widely used and cited guidelines proposed by Hu and Bentler (1999), RMSEA ≤ 0.06, CFI ≥ 0.95, and SRMR/RMR ≤ 0.08 indicate cutoff levels for accepting (good) model fit. Values of RMSEA between 0.06 and 0.08, CFI between 0.90 and 0.95, and SRMR 0.08 and 0.10 indicate adequate model fit. For the GFI, good fit is usually ≥ 0.95 (Schreiber et al., 2006), and adequate fit is > 0.90 to 0.95 (Shevlin & Miles, 1998). However, the GFI is now not recommended for evaluating model fit (Schreiber et al., 2006) as it is highly sensitive to sample size (Bollen, 1990) and the number of parameters estimated in the model (MacCallum & Hong, 1997). It is generally accepted that for evaluating model fit, most of the fit indices reported need to show good or acceptable levels (Hooper et al., 2008). For more detailed overviews of guidelines for evaluation model fit, the reader is referred to Hooper et al. (2008), and Schreiber et al. (2006).

An examination of past CFA studies of the SWBQ shows that the findings of only two studies can be interpreted as meeting standards for good global fit in terms of GFI and SRMR values for the four-factor model (Gomez & Fisher, 2005; Rowold, 2011). The fit indices reported in the original study by Gomez and Fisher (2003) met the good fit cutoff for the SRMR, but not for the GFI for the two groups examined, and neither the CFI nor RMSEA met the criteria for a good fit where used (Gouveia et al., 2012; Nunes et al., 2018). Additionally, the one-factor model (Gomez & Fisher, 2005; Rowold, 2011), the four-factor orthogonal model (Gomez & Fisher, 2005), and the higher-order model (Gomez & Fisher, 2003; Gouveia et al., 2012; Nunes et al., 2018) all failed to show good or even adequate fit. Taken together these findings indicate no, or at best weak support for the one-factor, four-factor orthogonal model, and higher-order factor models. While there was some support for the four-factor model, its support is at best mediocre, coming from only GFI and SRMR fit values. However, as noted earlier, GFI is not recommended for evaluating model fit. It can, therefore, be argued that CFA has yet to clearly establish the factor structure of the SWBQ.

As will be evident, our suggestion that there is little or no support for the one-factor, four-factor orthogonal, higher-order factor, and four-factor models are all based on CFA studies. Despite its wide use, in recent years the standard CFA approach (that was used in the CFA studies involving the SWBQ), often referred to as the independent cluster CFA model (ICM-CFA), has come under criticism (Marsh et al., 2009). The ICM-CFA is an a priori model in which items are specified to load only onto the designated factors, without any cross-loadings. Questions have been raised regarding the zero constraints placed on cross-loadings imposed in this model because it is highly restrictive and artificial, as items in a multidimensional measure are rarely pure indicators of their latent factors. Some degree of construct-relevant overlap or association with non-target but conceptually related factors is to be expected (Morin et al., 2016). This is likely to be the case with the SWBQ, as EFA studies of SWBQ items have shown significant and substantial cross-loadings (e.g., Elhai et al., 2018; Gomez & Fisher, 2003; Neves et al., 2018; Pong et al., 2020). Indeed, for the EFA study of the SWBQ involving older institutionalized adults in Portugal, Neves et al. reported support for a 3 factors: environmental, transcendental, and humanitarian. In terms of item content, the environmental and transcendental factors were comparable to the items proposed originally for the SWBQ (Gomez & Fisher, 2003). The humanitarian factor comprised items from the personal (2 items) and communal (4 items) factors proposed originally for the SWBQ.

Asparouhov and Muthén (2009) have developed the exploratory structural equation modeling (ESEM) with a targeted rotation that is especially useful for testing the factor structure of a multidimensional measure (Marsh et al., 2009), such as the SWBQ. The ESEM procedure allows testing of an a priori defined structure (like CFA) whist allowing nonzero cross-loadings (like EFA). This approach, therefore, overcomes a limitation of CFA while retaining its advantages (i.e., being model-based). As shown in Fig. 1, in an ESEM model the targeted symptoms load on their own designated factors as well as all other non-designated factors at values close (but not forced) to zero. Studies have demonstrated that the ESEM approach is superior to the EFA and CFA approaches for testing factor structure (Marsh et al., 2009, 2014). It can therefore be speculated that since some of the SWBQ items have shown cross-loadings, the application of the ESEM approach is more suitable for testing the factor structure of the SWBQ, and it could potentially reveal better support for the theorized SWBQ four-factor model. To date, the ESEM approach has not been applied to study the factor structure of the SWBQ, although there are clear advantages to taking this approach.

Related to the factor structure evaluation of measures, the traditional EFA and CFA procedures as expounded by Koenig and Al Zaben (2021) are now required by the Journal of Religion and Health for evaluating the factor structure of new scales and/or translated existing scales related to religion or spirituality and health. However, as noted earlier, CFA has come under criticism and the ESEM approach has been recommended by experts for evaluating factor structure (Asparouhov & Muthén, 2009; Marsh et al., 2009; Morin et al., 2016). Additionally, we also argue that CFA is not suitable (due to cross-loadings) for evaluating the factor structure of the SWBQ, and that the ESEM is suitable (as it accounts for cross-loadings). Consequently, our use of ESEM instead of CFA in the current study should not be interpreted as problematic for this journal.

### Aims of the Current Study

The aim of the current study was to use CFA and ESEM to evaluate the factor structure of SWBQ. In all, four different models were examined: a one-factor model (Model 1); a CFA four-factor oblique model (Model 2); an ESEM with four oblique factors (Model 3), and; a higher-order factor model (Model 4). These models are shown in Fig. 1.

For potentially optimum structural model(s), we also computed model-based reliabilities (omega; Zinbarg et al., 2005) for the different factors. Related to internal consistency it may be worth noting that past studies have provided strong support in terms of coefficient alpha for the four scales in the SWBQ, with values > 0.80 (Abhari et al., 2018; Fisher, 2010; Gomez & Fisher, 2003, 2005; Gouveia & Marques, 2012; Gouveia et al., 2009; Holder et al., 2010; Nunes et al., 2018; Rowold, 2011). Despite this, it is worth noting that compared to coefficient alpha, coefficient omega is considered a better unbiased estimate of internal consistency (Zinbarg et al., 2005).

We also examined criterion-related validities of the group factors for the potentially optimum model(s). This involved correlating a range of well-being measures with the SWBQ factors. As noted earlier, the review by de Jager Meezenbroek et al. (2012) found that only one (“developing joy in life”) of the 20 SWBQ items is confused with (health) well-being. Consequently, scores for the spirituality constructs in this measure and other health well-being measures will not be inflated due to tautology. Existing cross-sectional data show that all four domains in the SWBQ (personal, communal, environmental, and transcendental) are associated positively with happiness (Elhai et al., 2018; Gomez & Fisher, 2003; Holder et al., 2010), mental, physical, and emotional well-being (Rowold, 2011), life satisfaction (Elhai et al., 2018), and personal well-being (Nunes et al., 2018). Abhari et al. (2018) found this only for the personal domain. There are also longitudinal findings showing that the personal and communal domains uniquely and positively predicted subsequent happiness; the personal and transcendental domains uniquely and positively predict subsequent psychological well-being; and the personal and communal domains uniquely and negatively predict subsequent stress (Rowold, 2011).

Taken together, these findings indicate that cross-sectionally, all the four SWBQ domains (personal, communal, environmental, and transcendental) are associated with a range of psychological, emotional, mental, and physical well-being constructs, and that longitudinally, the personal, and to a lesser degree the communal and transcendental domains, are associated with better psychological well-being and less stress. In the current study, the criterion well-being variables used for testing the validity of the SWBQ domains were self-esteem, loneliness, and satisfaction with life. Given past findings (Abhari et al., 2018; Elhai et al., 2018; Gomez & Fisher, 2003; Holder et al., 2010; Nunes et al., 2018; Rowold, 2011), it can be argued that these psychological well-being variables are relevant for testing the validities of all four SWBQ domains.

In terms of predictions, we did not expect global fit support for the one-factor and higher-order factor models. As we were applying the more advanced ESEM model that allows cross-loadings, we expected some support for global fit for the ESEM four-factor oblique model. Concerning reliability, we expected support for the factors in the ESEM four-factor oblique model but made no predictions about the support for the validities of the factors in this model.

## Method

### Participants

The sample used for testing the factor structure and reliability of the SWBQ was from the general community and comprised 227 adults (women = 164; men = 63), ranging in age from 18 to 56 years. The sample used to examine the external validities of the factors in the preferred SWBQ model was a subsample of 102 individuals (women = 90; men = 12). For all participants together, the mean age was 26.07 years (SD = 5.15). The mean age for women was 26.64 years (SD = 8.80), and for men, it was 27.19 years (SD = 7.12). The gender groups did not differ significantly for age, *t* (225) = 1.14, *p* = 255. Overall, 78.8% of the sample was single, 18.2% were married, and the remaining 3% were separated, divorced, or widowed. In terms of the highest educational level attended, 66.5% had completed secondary education or a trade certificate, and the other 32.5% had or were completing a tertiary education program. In terms of employment, 42.9% were full-time employed, 32.1% were part-time employed or casual, and 25.0% were unemployed (including students) or retired.

### Measures

Participants completed a demographic sheet that sought information about their age gender, education, employment, and relationship status. The self-report questionnaire measures used in the current study were the Spiritual Well-Being Questionnaire (SWBQ; Gomez & Fisher, 2003), the Rosenberg Self-Esteem Scale (RSES; Rosenberg, 1965), the UCLA Loneliness Scale-Version 3 (UCLA LS3; Russell, 1996), and the Satisfaction with Life Scale (SWLS; Diener et al., 1985).

#### Spiritual Well-Being Questionnaire (SWBQ; Gomez & Fisher, 2003)

The current study used the identical version of the SWBQ that was used in the original SWBQ development and validation of the study. The SWBQ was described in the Introduction. Each of the 20 items in the SWBQ is rated on a five-point interval, ranging from 1 (*very low*) to 5 (*very high*), with higher scores indicative of higher spiritual well-being. In the current study, the internal reliability (alpha coefficient) for the personal, communal, environmental, and transcendental spiritual well-being subscales were 0.79, 0.80, 0.86, and 0.86, respectively.

#### Rosenberg Self-Esteem Scale (RSES; Rosenberg, 1965)

The 10-item RSES was used in the current study to measure dispositional self-esteem. Each item is scored on a four-point response interval ranging from 1 (*strongly agree*) to 4 (*strongly disagree*), with higher scores reflecting higher self-esteem. Example items in the RSES are “On the whole, I am satisfied with myself,” and “I take a positive attitude toward myself.” The current study used the total score (after reversing the scores for the reverse worded items), based on all 10 items, to measure self-esteem. The scales had demonstrated good reliability and validity (Rosenberg, 1965; Sinclair et al., 2010). The internal consistency (alpha coefficient) for the RSES in the current study was 0.89.

#### UCLA Loneliness Scale-Version 3 (UCLA LS3; Russell, 1996)

The 20-item UCLA LS3 was used to measure loneliness. Each item is scored on a four-point response interval that ranges from 1 (*never*) to 4 (*always*), with higher scores indicating higher levels of loneliness. Examples of items in the UCLA LS3 are “I have nobody to talk to,” and “I feel left out.” In the current study, we used the total score, based on all 20 items, to measure loneliness. The UCLA LS3 has demonstrated good reliability and validity (Russell, 1996). The internal consistency (alpha coefficient) for the sample in the current study was 0.86.

#### Satisfaction with Life Scale (SWLS; Diener et al., 1985)

The 5-item SWLS was used to measure overall satisfaction with life. Each item is scored on a seven-point response interval ranging from 1 (*strongly disagree*) to 7 (*strongly agree*), with higher scores indicating greater overall life satisfaction. Examples items are “In most ways, my life is close to my ideal,” and “The conditions of my life are excellent.” The current study used the total score, based on all 5 items, as the measure of life satisfaction. The SWLS has demonstrated good reliability and validity (Diener et al., 1985; Pavot & Diener, 2009). The internal consistency (alpha coefficient) for the SWLS in the current study was 0.89.

### Procedure

Prior to data collection, ethics approval was obtained from University of Ballarat’s (now Federation University) Human Ethics Committee. All participants were from the general community in the state of Victoria, Australia. They were recruited in locations where many individuals congregate, such as shopping centers, and sporting, recreational, and social clubs and organizations. The recruitment, conducted by research assistants, involved approaching random and directly potential participants in these centers. The research assistants introduced themselves, then briefly explained the background of the study, including the research procedure, and then invited them to participate in the study. Potential participants were informed that the research study was aimed at examining how individuals score on various aspects related to spirituality and how these aspects are related to health. Those who expressed interest and willingness were given an envelope with a plain language statement about the study, an informed consent form, and a set of questionnaires. The plain language statement indicated the need to complete the questionnaires by themselves. All consenting participants who completed the questionnaires returned them either by handing them back directly to the research assistants or in prepaid envelopes supplied to them.

In all, around 500 questionnaires were distributed with the SWBQ included. Of these, 300 envelopes also included the SWLS (Diener et al., 1985), the UCLA LS3 (Russell, 1996), and RSES (Rosenberg, 1965). Two hundred and twenty-seven completed ratings for the SWBQ were returned, resulting in a return rate of approximately 45.4%. Of these, 102 participants also returned completed ratings for the SWLS, UCLA LS3, and RSES (34% of the number distributed). Because of ethical restrictions, information from those who did not participate was not obtained.

### Statistical Analysis

Regarding statistical power, the sample size in the current study is above the level recommended by some researchers for factor analyses involving 20 indicator items (i.e., a minimum sample size of 20 × 10 = 200; Myers et al., 2011). Additionally, we used Soper’s (2022) software for computing sample size requirements for the CFA model. The anticipated effect size was set at 0.3 (by convention, values of 0.1, 0.3, and 0.5 are small, medium, and large, respectively), power at 0.8, the number of latent variables at 4, the number of observed variables at 20, and the probability at 0.05. The analysis recommended a minimum sample size of 137. Our sample size (*N* = 227) was well above this recommendation. All statistical analyses were conducted using M*plus* Version 7.3 (Muthén & Muthén, 2012). Maximum likelihood (ML) extraction was used. The ESEM model in the study was conducted using geomin (i.e., oblique) rotation. In the ESEM, items were loaded on the designated factors, and cross-loadings were “targeted,” but not forced, to be as close to zero as possible.

To establish the optimum model, we followed four steps that involved (1) global model fit criterion, (2) clarity criterion, (3) reliability, and (4) external validity criteria. In step 1, we selected all good global fitting models as potential good models for the SWBQ. Global fit was evaluated using the approximate fit indices provided in M*Plus* (i.e., root mean square error of approximation [RMSEA], Tucker–Lewis index [TLI], comparative fit index [CFI], and standardized root mean square residual [SRMR]). Of the approximate fit indices reported in M*plus*, Hu and Bentler (1998) have recommended a two-index approach for evaluating model fit that includes good fit in terms of the SRMR value and either the TLI, CFI, or RMSEA. For the current study, a globally good fitting model was defined a priori using this recommendation. According to the widely used and cited guidelines proposed by Hu and Bentler (1999), RMSEA ≤ 0.06, CFI and TLI ≥ 0.95, and SRMR ≤ 0.08 indicate cutoff levels for accepting good model fit. Values of RMSEA between 0.06 and 0.08, CFI between 0.90 and 0.95, and SRMR 0.08 and 0.10 indicate adequate model fit. We also computed the Akaike Information Criterion (AIC) for all models. When compared to other models, smaller AIC values indicate a better fitting parsimonious model. The difference in the fit between nested models was examined using differences in the chi-square test together with the difference in AIC values.

In step 2, the potentially good models selected in Step 1 were examined for factor clarity. For this, the pattern (significance) of factor loadings and cross-loadings (when appropriate) were examined. Cross-loading was defined in terms of an item loading significantly on two or more factors. To meet the clarity criterion, an ideal model should have 100% significant target items and 0% significant non-target items. The model with more significant loadings on the designated factors and fewer significant cross-loadings on non-designated factors was selected as being the more clearly defined model.

In Step 3, the omega reliabilities of the factors selected in Step 2 were examined (Arias et al., 2018; Zinbarg et al., 2005). Ranging from 0 to 1, higher values for these indices indicate better reliabilities (Brunner et al., 2012). Reise et al. (2013) have suggested that values of at least 0.50 with values of at least 0.75 are preferred for meaningful interpretation of a scale.

In Step 4, the external validities of the factors selected in Step 2 were examined. Given that apart from the transcendental dimension, the other SWBQ dimensions—especially the personal and communal dimensions—can be considered as measures of mental health more so than religion or spirituality, attempts to examine the relationship between the personal and communal dimensions with mental health will be confounded. A reviewer suggested that establishing the relationships of the non-religious SWBQ dimensions with mental health measures should be conducted separately, and not be included altogether in the model at once or combined into a single score. Also, the religious dimension (i.e., the transcendental dimension) should be modeled in such a way that it partials out the shared variance it has with the other dimensions since the other dimensions share variance with the external mental health outcome variables being examined. Thus, the relationships of the SWBQ personal, communal, and environmental dimensions with our external criterion variables (satisfaction with life, loneliness, and self-esteem) were examined in the study using correlation analysis, whereas the relationships of the SWBQ transcendental dimension with our external criterion variables were examined using regression analysis in which the external criterion variables were regressed on all four SWBQ dimensions simultaneously using an SEM framework. Support for their external validity was assumed if the factors (that demonstrated acceptable reliabilities) were associated with one or more of the external variables in the theoretically expected direction.

## Results

### Step 1: Examining Global Fit of Model Tested

The fit values for the 4 models tested in the study are shown in Table 1. Model 1 showed poor fit in terms of the RMSEA, CFI, TLI and SRMR values. Model 2 (four-factor oblique mode) and Model 4 (higher-order factor model) showed adequate fit in terms of their RMSEA, CFI, and TLI values, and good fit in terms of SRMR values. Model 3 (ESEM model with specific factors for personal, communal, environmental, and transcendental) showed good fit in terms of its CFI and SRMR values, and adequate fit in terms of its RMSEA and TLI values. Of the 4 models tested, only Model 3 (ESEM model with specific factors for personal, communal, environmental, and transcendental) was deemed a potentially good model, based on the a priori criteria adopted for the study (good fit for the SRMR value and either the TLI, CFI, or RMSEA value). For Model 3, the SRMR value was good (0.028), as was its CFI value (0.952). The RMSEA and TLI values were adequate (0.074 and 0.929, respectively). Also, Model 3 had the lowest AIC value, and it showed better fit than Model 2 [Δ*χ*^2^ (Δdf = 48) = 112.01, *p* < 0.001], and Model 4 [Δ*χ*2 (Δdf = 50) = 117.45, *p* < 0.001]. Thus, only Model 3 was examined for factor clarity, reliabilities, and validities.

**Table 1:** Fit of the factor models of the SWBQ (*N* = 227)

Models (M)	*df*	*χ*^2^	RMSEA		CFI	TLI	SRMR	AIC
			Estimate	90% CI				
M1. One factor	170	1777.09	.204	[.196, .213]	.465	.403	.146	12,036
M2. Four oblique factors	164	372.59	.075	[.065, .085]	.931	.920	.**057**	10,644
M3. ESEM / Four oblique factors	116	260.58	.074	[.062, .086]	**.952**	.921	**.028**	10,628
M4. Higher-order/four factors	166	378.03	.075	[.065, .085]	.929	.919	**.061**	10,645

*χ*^2^ maximum likelihood *χ*^2^, *RMSEA* root mean square error of approximation; *CFI* comparative fit index; *TLI* Tucker–Lewis index; *SRMR* standardized root mean square residual; *AIC* Akaike Information Criterion; *ESEM* exploratory structural equation modeling. Underlined and bold are fit values meeting cutoff scores for good model fit. Underlined and not bold are fit values meeting cutoff scores for adequate model fit

All *χ*^2^ values were significant (*p* < .01)

### Step 2: Examining the Item-Factor Loadings in Model 3

Table 2 shows the factor loadings for Model 3. It also includes a summary of the number of targeted factor loadings and cross-loadings in these models. As shown in Table 2, all designated items loaded significantly on their designated factors. With 15 potential significant cross-loadings, the numbers of significant cross-loadings for the personal, communal, environmental, and transcendental factors were 1, 4, 2, and 2, respectively. Thus although 9 cross-loadings were present in Model 3, all the factors in this model were reasonably well defined, and none of the cross-loadings were above the salient level, defined in terms of loadings above 0.45 (Tabachnick & Fidell, 2007). Thus, we took this as meeting the clarity criterion. As shown in Table 2, the correlations between the factors in this model were all significant (ranging from 0.23 to 0.63). Based on the suggestion that correlations < 0.50 are low (Moore et al., 2013), the correlations for transcendental with personal, communal with environmental, and communal with environmental can be considered low. The correlations between personal and communal, and personal and environmental can be considered moderate.

**Table 2:** Completely standardized factor loadings, reliabilities, and correlations of the factors in the ESEM model

#	Item	P	C	E	T
5	Developing a sense of identity	.85***	− .14	.00	.01
9	Developing self-awareness	.60***	.11	.00	− .09
14	Developing joy in life	.62***	.19	− .14	.03
16	Developing inner peace	.50**	.01	.23**	.07
18	Developing meaning in life	.32*	.20*	.10	.17**
1	Developing a love of other people	.03	.54***	.00	− .02
3	Developing forgiveness toward others	.08	.52***	.08	.18***
8	Developing trust between individuals	.29**	.37***	.06	− .10
17	Developing respect for others	.08	.78***	.02	.01
19	Developing kindness toward other people	− .09	.84***	.02	− .04
4	Developing connection with nature	.11	− .05	.79***	.03
7	Developing awe at a breathtaking view	− .13	.19*	.60***	.03
10	Developing oneness with nature	− .01	− .11*	.93***	.04
12	Developing harmony with the environment	.08	.00	.86***	− .05
20	Developing a sense of magic in the environment	− .04	.14*	.67***	− .03
2	Developing a personal relationship with God	.05	− .01	.05	.85***
6	Developing worship of the Creator	.02	− .02	− .07	.93***
11	Developing oneness with God	− .02	− .04	.07**	.95***
13	Developing peace with God	.02	− .01	.02	.92***
15	Developing prayer life	− .03	.09	− .08	.85***
*Reliability*					
Omega (*ω*)		.724	.767	.883	.956
*Correlations*					
Personal		–	.63***	.60***	.30***
Communal			–	046***	.23***
Environmental				–	.36***
Transcendental					–

*ESEM* exploratory structural equation modeling; *P* Personal; *C* Communal; *E* Environmental; *T* Transcendental. Boldface values indicate factor loadings in the primary dimension

*p < .05. **p < .01. ***p < .001

### Step 3: Examining Reliabilities of the Factors in Model 3

The omega values for the factors in Model 3 are shown in Table 2. As shown in the table, the *ω* values for the personal, communal, environmental, and transcendental factors were 0.724, 0.767, 0.883, and 0.956, respectively. As these values are above the value proposed for meaningful interpretation of a factor (*ω* values > 0.50; Reise et al., 2013), the reliabilities of all the factors in this model can be interpreted as adequate.

### Step 4: Examining Validities of the Factors in Model 3

Table 3 shows the correlations of all the external criterion variables (life satisfaction, loneliness, and self-esteem) by the factors in Model 3. As shown, and as theoretically expected, life satisfaction was correlated positively with communal, and loneliness was correlated negatively with communal. However, all other correlations were not significant. These findings indicated some support for the external validity of the communal factor, and no support for the external validity of the personal, environmental and transcendental factors. Thus, there was incomplete support for the validities of all the factors in Model 3.

**Table 3:** Standardized coefficients for the associations of satisfaction with life, loneliness, and self-esteem and the external criterion variables in the ESEM model (*N* = 102)

	Satisfaction with life	Loneliness	Self-esteem
Personal	0.10	− 0.10	0.21
Communal	0.28*	− 0.43***	0.00
Environmental	0.07	0.04	0.15
Transcendental	− 0.02	0.12	− 0.17

The relationships for personal, communal, and environmental dimensions are correlation coefficients. The relationships of transcendental dimension are path coefficients in which the external criterion variables were regressed on all four SWBQ dimensions simultaneously.*ESEM *exploratory structural equation modeling.

*p < .05. **p < .01. ***p < .001

## Discussion

The major aim of the current study was to establish the optimum model for the SWBQ. In all, we tested four different models: one-factor CFA model (Model 1), four-factor oblique CFA model (Model 2), ESEM with four group factors (Model 3), and higher-order CFA factor with four primary factors (Model 4). The four group factors in all models were personal, communal, environmental, and transcendental.

The findings showed unacceptable fit for the one-factor model (Model 1), Model 3 (ESEM model with group factors for personal, communal, environmental, and transcendental) was deemed as a potentially good model, based on the a priori criteria adopted for the study. The four factors in Model 3 were reasonably well defined, with few anomalies in factor loadings. Also, all the factors in this model were supported in terms of their reliabilities. Concerning validity, although we have interpreted our findings as supportive for the communal well-being domain, but not for the personal, environmental, and transcendental well-being domains in Model 3, there is a need for caution with this interpretation. As all the criterion-related variables used in the external validation analyses (life satisfaction, loneliness, and self-esteem) focused on psychological well-being, our findings could be highlighting potential tautological problems in our external validation findings. Although based on past data, we argued that the variables that we selected for testing the externality validities of the SWBQ domains were appropriate, this may not have been the case. If so, the study was limited in its ability to provide a clear and credible test of the criterion validities of all SWBQ domains. Consequently, our findings that showed a lack of support for the external validities of the personal, environmental, and transcendental domains in Model 3 cannot be taken as grounds for rejecting Model 3. In contrast to model 3, our findings showed only adequate fit for the theorized four-factor oblique model (Model 2) and the higher-order factor model (Model 4). Notwithstanding this, the findings can be interpreted as providing some tentative support for model 2 and 4.

Using the same cutoff as used in the current study to ascertain fit levels, the fit for the one-factor model in past studies (Gomez & Fisher, 2003; Nunes et al., 2018; Rowold, 2011) was reinterpreted (in the Introduction) as indicating unacceptable fit. This was also the case for the higher-order factor model (Nunes et al., 2018; Rowold, 2011) and the four-factor oblique model (Gomez & Fisher, 2003, 2005; Gouveia et al., 2012; Rowold, 2011). These findings are consistent with the findings in the current study which showed unacceptable fit for the one-factor, higher-order factor, and four-factor oblique models. Despite these similarities, the findings in the current study also extend existing findings. This is because, unlike past studies, the current study applied ESEM modeling procedures (not applied previously). Thus, the findings involving Model 3 are new. As already noted, this model showed better fit than all the models tested in previous studies (Models 1, 2, and 4).

In conclusion, the findings in the current study do not support the view that the SWBQ has proven construct validity in terms of a higher-order factor structure with groups factors for personal, communal, environmental, and transcendental well-being factors (de Jager Meezenbroek et al., 2012). Concerning Fisher’s (1998) spiritual well-being model, the lack of support for the general factor in Model 4 does not necessarily mean that Fisher’s hierarchical multidimensional model of spiritual well-being is invalid. The problem is more likely to lie in the inability of the current version of SWBQ to cleanly measure the separate domains.

### Study Limitations

The findings and interpretations made in this study need to be viewed with several limitations in mind. First, as all the data in the current study were obtained using self-ratings questionnaires, the findings may be biased by common method variance. Second, as this was a cross-sectional study, causal relations cannot be inferred. Third, the sample comprised a convenience sample and was not a random sample. Thus, the generalizability of our findings is limited. However, it may be worth noting that virtually all studies involving the SWBQ and other spiritual well-being measures (Gomez & Fisher, 2003) have also used convenience samples. Fourth, as there is a female to male ratio of 2.6:1 in the CFA and ESEM analysis, the findings may be seen as biased to females and therefore not applicable to adults in general. Fifth, although our analysis indicated that our sample size (*N* = 227) for the CFA and ESEM analyses provided sufficient power for our study, it is worth noting that some researchers have suggested much larger sample sizes for CFA models. For instance, Boateng et al. (2018) suggested that for CFA models, sample sizes ≤ 100 = poor; > 100 to ≤ 200 = fair; > 200 to ≤ 300 = good; > 500 = very good; and > 1000 = excellent. Thus, although our sample size could be considered good for the CFA and ESEM analysis, it could be seen as not sufficiently adequate. Related to sample size, as the SEM conducted for establishing the criterion validity of the SWBQ factors was only 102, this could be considered too low for reliable estimates, thereby adding another reason for caution when interpreting the external validity findings. Sixth, for evaluating external validity, we used only a limited number of external variables. This may have limited our ability to provide a credible test for external validity.

## Conclusions

Given the limitations noted above, the generalizability of the findings in the study can be questioned. Overall, the findings provided tentative support for a four-factor ESEM oblique model. However, the study design did not allow us to clearly assess the validity of the factors in this model. If so, it means that there is a need to undertake future studies to establish their criterion validity if the goal is for this measure to serve as a valid research measure for measuring spiritual well-being, as defined in Fisher’s model. Notwithstanding this, although the psychometric review by de Jager Meezenbroek et al. (2012) concluded that only one SWBQ item was confused with general well-being, this may not necessarily imply that the items are not confounded with mental and psychological health. Thus, the findings here cannot be generalized to more distinctively “spiritual” or religious dimensions that are not contaminated by indicators of mental health. Additionally, although we adopted Model 3 as our preferred model, this model had only a small advantage over Models 2 and 4 in the study. Even though Models 2 and 4 did not meet our a priori criteria for a good model (good fit values in at least two of the four fit indices), both these models showed adequate fit. It is therefore conceivable that these models would potentially meet our a priori criteria in future replication studies involving other sample groups. Consequently, it will be useful for readers to keep this in mind when viewing the conclusions and interpretations made in this study.

Clearly, there is a need for more studies in this area, controlling for the limitations noted here. For this, the current study has demonstrated a useful and advanced methodological approach (ESEM) for future studies. As a concluding remark, it is important to note that the findings reported in the current paper are limited to Australia. Further research is needed to establish these findings in other countries and regions of the world where cultural and religious factors may influence findings. In this respect, we note that there are three potential models that warrant further detailed investigation with a larger, more representative population and additional validation measures.
